# Prognostic model of immune checkpoint inhibitors combined with anti-angiogenic agents in unresectable hepatocellular carcinoma

**DOI:** 10.3389/fimmu.2022.1060051

**Published:** 2022-12-01

**Authors:** Xiaomi Li, Wei Sun, Xiaoyan Ding, Wei Li, Jinglong Chen

**Affiliations:** Department of Cancer Center, Beijing Ditan Hospital, Capital Medical University, Beijing, China

**Keywords:** hepatocellular carcinoma, immune checkpoint inhibitors, anti-angiogenic agents, predictive model, platelet to lymphocyte ratio, alpha-fetoprotein

## Abstract

**Background:**

The combination of immune checkpoint inhibitors (ICIs) and anti-angiogenic agents has shown promising efficacy in unresectable hepatocellular carcinoma (HCC), but until now no clinical prognostic models or predictive biomarkers have been established.

**Methods:**

From 2016 to 2021, a total of 258 HCCs treated with ICIs and tyrosine kinase inhibitors (TKIs) were retrospectively enrolled, as the study cohort. Patients’ baseline data was extracted by least absolute and shrinkage selection operator (LASSO) and Cox regression. Finally, a prognostic model in the form of nomogram was developed. Model performance was assessed in terms of discrimination, calibration, and clinical utility. A 5-fold cross-validation was used to evaluate the internal repeatability of the model. In addition, the patient cohort was divided into three subgroups according to nomogram scores. Their survivals were estimated by Kaplan-Meier methods and the differences were analyzed using log-rank tests.

**Results:**

Seven clinical parameters were selected: Eastern Cooperative Oncology Group performance status (ECOG PS), combination of transarterial chemoembolization (TACE), extrahepatic metastasis (EHM), platelet to lymphocyte ratio (PLR), alanine aminotransferase (ALT), alpha-fetoprotein (AFP), and Child-Pugh score. The model had an area under the curve (AUC) of 0.777 at 1 year and 0.772 at 2 years. Receiver operating characteristic (ROC) curve, calibration curve and decision curve analysis (DCA) showed that the discrimination, consistency and applicability of the model were good. In addition, cross-validation validated the discrimination of the model, and the C index value of the model is 0.7405. The median overall survival (OS) of the high-, medium- and low-risk subgroups was 7.58, 17.50 and 53.17 months, respectively, with a significant difference between the groups (P < 0.0001).

**Conclusion:**

We developed a comprehensive and simple prognostic model for the combination of ICIs plus TKIs. And it may predict the efficacy of the combination regimen for unresectable HCC.

## Introduction

Primary liver cancer is ranked the sixth of cancer morbidity and the third of cancer mortality worldwide (1). And hepatocellular carcinoma (HCC) is the main pathological type of liver cancer (2). HCC has a poor prognosis, possibly due to untimely diagnosis and complex tumor microenvironment (3, 4). Sorafenib was the first approved front-line tyrosine kinase inhibitor (TKI) for unresectable HCC (5, 6). In recent years, four other TKIs (lenvatinib, regorafenib, cabozantinib, and ramucirumab) have been used for first-line or second-line treatment of HCC (7–10). While immune checkpoint inhibitors (ICIs) have shown clinical prospects in other cancers, these programmed cell death protein 1 (PD-1) monoclonal antibodies including nivolumab, pembrolizumab, camrelizumab and sintilimab, are approved in advanced HCC based on clinical trials (11–14). In the light of the synergic efficacy for the combination of anti-angiogenic TKIs or monoclonal antibody plus ICIs, the combination regimens have been validated and approved (15, 16). More notably, IMbrave150 and ORIENT-32 showed that the combination of ICIs and anti-angiogenic agents resulted in longer overall survival (OS) of 19.2 months and progression-free survival (PFS) of 6.9 months compared with mono-sorafenib (17, 18). Though with the increasing evidence for ICIs plus TKIs in advanced HCC, the prognosis of advanced HCC varies a lot and clinical predictive biomarkers are limited (15). Considering that some patients could not benefit at all, they would be screened by the prognostic model to further guide clinical choice.

As for the prognostic biomarkers, unlike other malignancy, tumor mutation burden (TMB) and programmed cell death protein ligand-1 (PD-L1) expression seems less predictive in HCC (15, 19, 20). The diversity of response to treatment and the potential severe adverse events makes the discovery of biomarkers of great clinical importance. Radiomics nomograms have achieved good predictive performance, with an area under the receiver operating characteristic (ROC) curve (AUC) of 0.880, but the acquisition and analysis of images often require professional radiologists and professional equipment (21, 22). Two radiomics models involved a sample size of less than 60 and uncertain treatment regimens, either mono-ICIs or the combination of anti-angiogenic agents plus ICI. Genomics utilizes tumor tissue for testing and it is invasive (23–25). Although these clinical predictive models are convenient to use, none of the current models involve clinical inflammatory factors (15). Indeed, the tumor microenvironment is closely related to the prognosis of HCC, and neutrophil-to-lymphocyte ratio (NLR), platelet-to-lymphocyte ratio (PLR), lymphocyte-to-monocyte ratio (LMR), prognostic nutritional index (PNI), and systemic immune inflammatory (SII) index are representative systemic inflammatory markers (26–29). The comprehensive model including the above factors could be developed, thus, the patients with poor prognosis will be promptly identified and invalid treatment might be avoided.

Therefore, this study retrospectively analyzed unresectable HCC treated with ICIs combined with TKIs. The predictive factors affecting patient survival were explored and a prognostic model for advanced HCC in Chinese population was developed, in whom HBV-infection was the main etiology.

## Methods

### Patients and data collection

Patients with unresectable HCC were retrospectively enrolled, who received PD-1 inhibitors and anti-angiogenic agents at Beijing Ditan Hospital, Capital Medical University from 2016 to 2021. The study conformed to the 1975 Declaration of Helsinki and has been approved by the Ethics Review Committee of Beijing Ditan Hospital, Capital Medical University. All patients provided written informed consent.

Inclusion criteria for this study included: (1) aged ≥ 18 years; (2) HCC diagnosed by imaging or pathological examination; (3) Child-Pugh class A or B; (4) Eastern Cooperative Oncology Group performance status (ECOG PS) < 2; (5) BCLC stage C or B unable to tolerate radical treatment. Meanwhile, the following patients were excluded: (1) combined with a history of other malignancy outside HCC or metastatic liver tumor; (2) pregnant or lactating women; (3) severe organ or blood dysfunction; (4) abdominal computed tomography (CT) and/or magnetic resonance imaging (MRI) were not performed. (5) loss of follow-up.

Patients’ clinical data were queried by electronic medical records, mainly including age, gender, ECOG PS, etiology (“HBV”or”HCV”or”other”), previous and present treatment (surgery, ablation, transarterial chemoembolization (TACE) and treatment line), Child-Pugh class and BCLC stage. In addition, imaging data (tumor number, tumor size, portal vein tumor thrombus (PVTT), extrahepatic metastasis (EHM)) and laboratory parameters (peripheral blood count, liver function, and tumor marker AFP) were collected before the start of treatment, and NLR (absolute neutrophil count/absolute lymphocyte count), PLR (absolute platelet count/absolute lymphocyte count), LMR (absolute lymphocyte count/absolute monocyte count), PNI (serum albumin + 0.5 * absolute lymphocyte count), and SII (absolute neutrophil count * platelet count/absolute lymphocyte count) were calculated.

### Treatment and follow-up

All patients were treated with ICIs, and the selected PD-1 inhibitors mainly included camrelizumab (Hengrui Medicine, China) and sintilimab (Innovent Biologics, China), 200 mg intravenously every three weeks. In addition, the above patients could be included in this study whether they were TKI ‐ naïve or TKI ‐ experienced. The TKIs involved were sorafenib (Bayer Pharma AG, Germany) 400-800 mg/day orally; lenvatinib (Eisai Co., Ltd., Japan) 8 mg/day for body weight < 60 kg and 12 mg/day orally for body weight ≥ 60 kg; and regorafenib (Bayer Pharma AG, Germany) 160 mg/day orally every 4 weeks 1-3. Patient management was to modify dose and interrupt treatment according to local drug regulations.

Response to treatment and questioning survival status were assessed every 6-12 weeks after the start of treatment, and medication modifications were documented.

### Model development and validation

Histograms were plotted to view the data distribution, and logarithmic transformation was performed for continuous variables with a clearly skewed distribution. Alternative predictors were screened by least absolute shrinkage and selection operator (LASSO) regression, and then independent predictors were selected by multivariate Cox regression (30). Based on the above results, a nomogram was constructed as the final model. Model performance was then assessed by ROC curve, calibration curve, and decision curve analysis (DCA). ROC curve measures the discriminatory power of the model, and AUC value quantitatively describes discrimination, with 0.5 indicating no discriminatory power and 1 indicating the best discriminatory power (31). The consistency between the model predicted risk and the actual risk is represented by the calibration curve, and the closer to the slope line, the better the calibration (32). DCA curves assess the clinical utility of the model, with greater area under the curve having better applicability (33). To test the reproducibility of the model, we intend to use a 5-fold cross-validation. We randomly selected 80% of patients as the training set, and the rest as the validation set, calculate AUC value, repeat 1000 times, and record the results in box plot and scatter plot. A total score was calculated for each patient based on the nomogram assigned ratio and divided into three risk groups: low, medium, and high using X-tile software.

### Statistical analysis

All statistical analyses were implemented by R software (version 4.1.1, Chicago, IL). Quantitative data were presented as median (range), while qualitative data were presented as number (percentage). OS was calculated from the start of treatment until death or last follow-up, survival curves were plotted by Kaplan-Meier method, and median survival (mOS) and 95% confidence interval (95% CI) were reported. Log-rank test was used when comparing different risk groups and P < 0.05 was considered statistically significant. The software packages involved in this study were “ggplot2”, “survminer”, “survival”, “glmnet”, “rms”, “riskRegression”, “dcurves”, “caret”, “nomogramFormula” and “dplyr”.

## Results

### Patient characteristics

**Figure 1:**
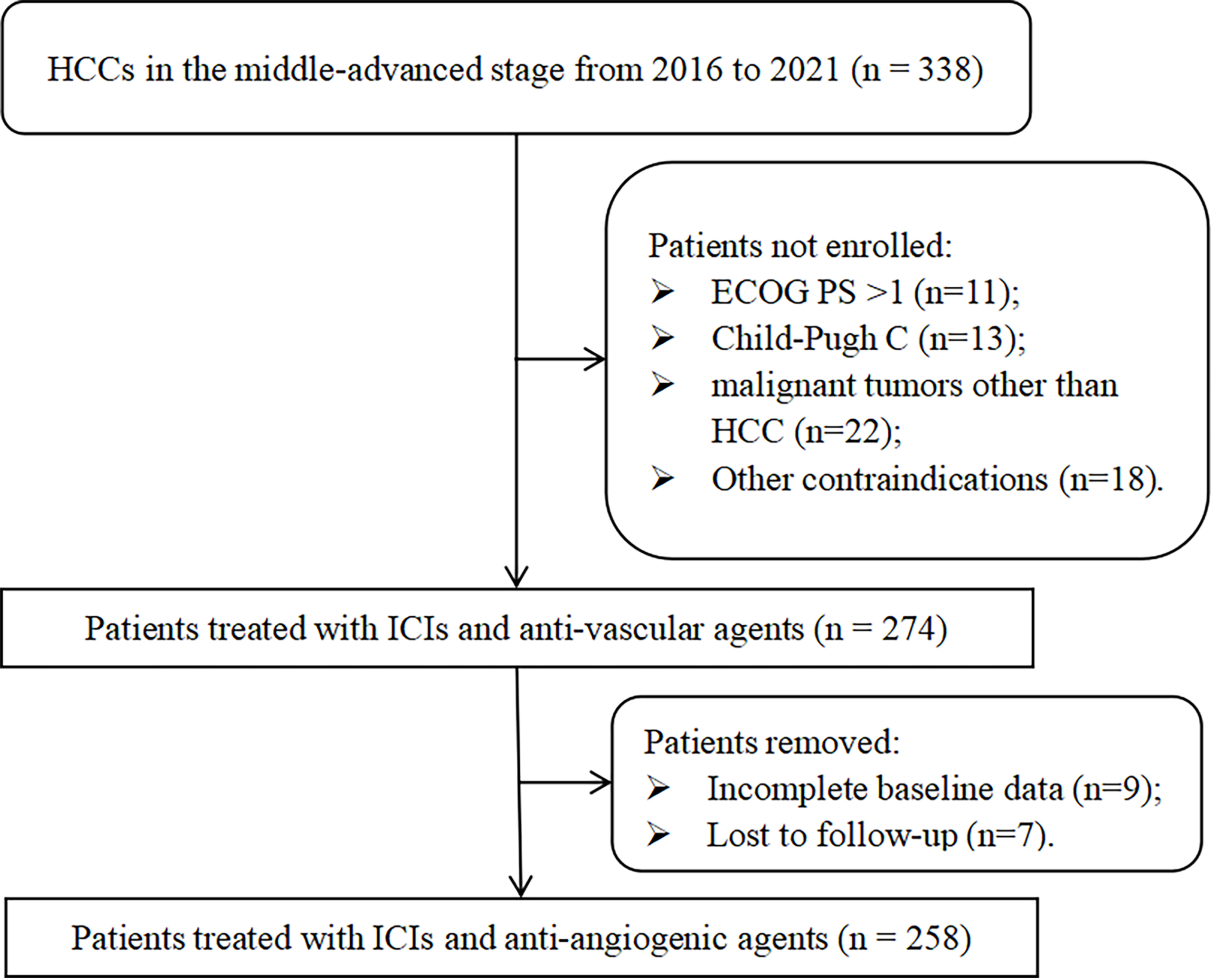
Flowchart of the study.

**Figure 2:**
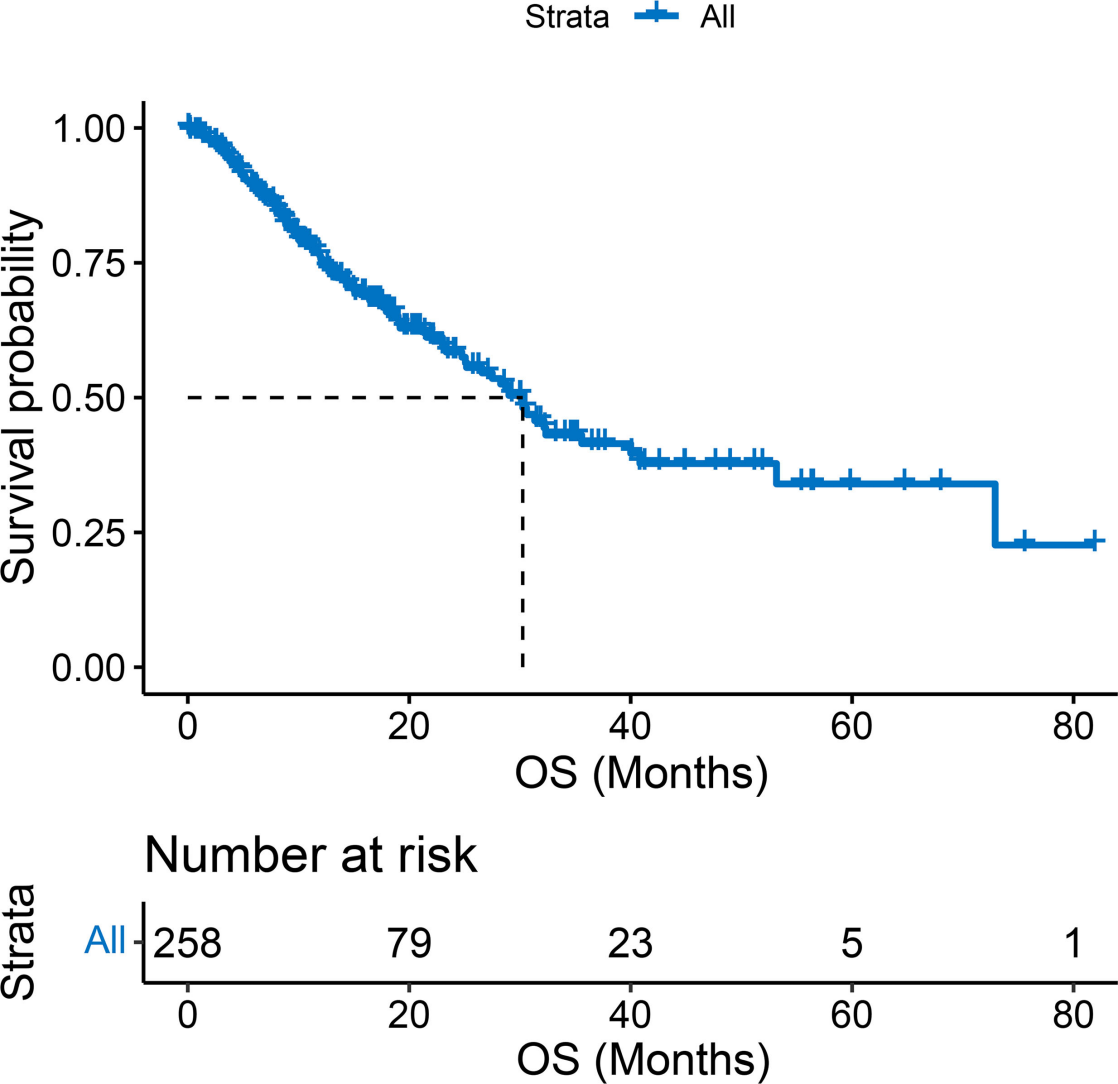
Kaplan-Meier curves of overall survival (A) in whole population.

Two hundred and fifty-eight patients treated with ICIs and TKIs were recruited retrospectively in this study (**Figure 1**). Patients’ baseline characteristics are shown in **Table 1**, with a median age of 59 (range, 51-65) years, 39 (15.1%) women and 219 (84.9%) men. And the majority of HCC was caused by HBV infection (n = 230, 89.1%). Ninety-seven (37.6%) patients had ECOG PS of 1, 157 (60.9%) had intrahepatic tumors > 3, 157 (60.9%) had maximum tumor diameter > 5 cm, and 76 (29.5%) had Child-Pugh class B. Overall, 195 (75.6%) patients were in BCLC stage C and had indications for PD-1 inhibitors and anti-angiogenic agents. In addition, although one-quarter of patients were in the middle stage of HCC, progression after TACE required the combination of systemic therapy. Other treatment history before ICI varied, with 40 (15.5%) and 109 (42.2%) cases receiving surgery and ablation, respectively, and 225 (87.2%) cases had combined TACE, 109 (42.2%) patients were first-line and the rest were later-line. In addition, 137 (53.1%) patients had PVTT and 101 (39.1%) had EHM. Until the last follow-up, a total of 97 (37.6%) patients had died, with a mOS of 30.2 (95% CI, 25.1 – 40.8) months, and their survival curves are shown in **Figure 2**.

**Table 1:** Patient baseline characteristics.

Characteristic	Value (n=258)
Age (years)	59.0 [51.0, 65.0]
NLR	2.9 [2.0, 4.4]
PLR	120.6 [82.6, 173.6]
LMR	2.6 [1.8, 3.5]
PNI	37.5 [33.7, 40.8]
SII	349.4 [204.4, 688.0]
AFP (ng/mL)	139.8 [6.8, 2000.0]
ALT (U/L)	31.4 [20.2, 58.4]
AST (U/L)	40.0 [29.9, 66.5]
A/G	1.2 [1.0, 1.4]
Sex, n(%)	
Male	219 (84.9)
Female	39 (15.1)
Etiology, n (%)	
HBV	230 (89.1)
HCV	11 (4.3)
Others	17 (6.6)
ECOG PS, n (%)	
0	141 (54.7)
1	117 (45.3)
Number, n (%)	
≤3	101 (39.1)
>3	157 (60.9)
Size, n (%)	
≤5cm	101 (39.1)
>5cm	157 (60.9)
PVTT, n (%)	
0	121 (46.9)
I	22 (8.5)
II	52 (20.2)
III	42 (16.3)
IV	21 (8.1)
EHM, n(%)	101 (39.1)
Prior therapy, n(%)	
Recection	40 (15.5)
Ablation	109 (42.2)
Combined with TACE, n(%)	225 (87.2)
Cycles ≤3	156 (60.5)
Cycles >3	69 (26.7)
Treatment line	
First line	165 (64.0)
Later line	93 (36.0)
Child-Pugh score, n (%)	
5	93 (36.0)
6	89 (34.5)
7	43 (16.7)
8	22 (8.5)
9	11 (4.3)
ALBI, n (%)	
1	49 (19.0)
2	177 (68.6)
3	32 (12.4)
BCLC stage, n (%)	
B	63 (24.4)
C	195 (75.6)

NLR, neutrophil-to-lymphocyte ratio; PLR, platelet-to-lymphocyte ratio; LMR, lymphocyte-to-monocyte ratio; PNI, prognostic nutritional index; SII, systemic immune-inflammatory; AFP, alpha-fetoprotein; ALT, alanine aminotransferase; AST, aspartate aminotransferase; A/G, albumin/globulin ratio; HBV, hepatitis B virus; HCV, hepatitis C virus; ECOG PS, Eastern Cooperative Oncology Group performance status; PVTT, portal vein tumor thrombosis; EHM, extrahepatic metastasis; TACE, transarterial chemoembolization; ALBI, albumin-bilirubin; BCLC, barcelona clinic liver cancer.

### Model development and validation

**Figure 3:**
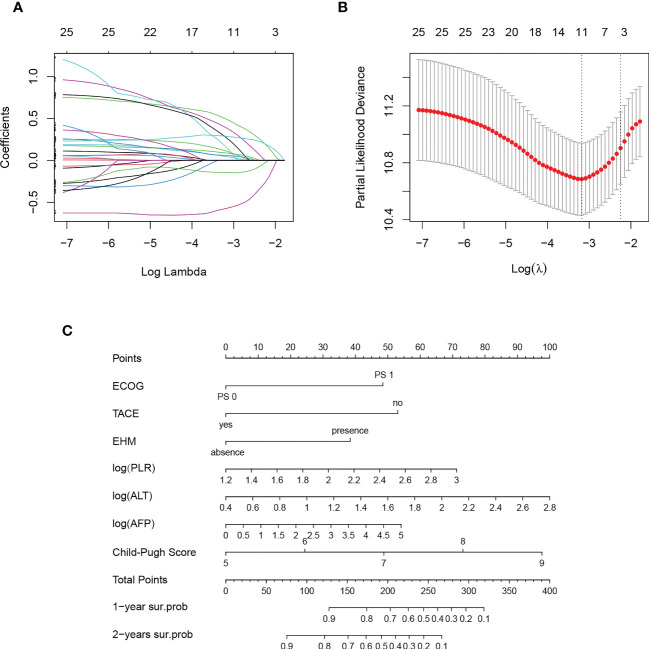
Result of Lasso regression **(A, B)** and nomogram for combination regimen for unresectable hepatocellular carcinoma **(C)**.

Histograms were used to view the data distribution of continuous variables. NLR, PLR, SII, alanine aminotransferase (ALT), aspartate aminotransferase (AST) and AFP showed a significantly skewed distribution, and logarithms were taken for the above factors. While other variables (age, LMR, PNI and albumin/globulin ratio (A/G)) were not converted. Eleven variables were selected by lambda values corresponding to partial Likelihood Deviance in Lasso regression: ECOG PS, the combination of TACE, tumor number, PVTT, EHM, A/G, log (PLR), log (ALT), log (AFP), ALBI class and Child-Pugh score (**Figures 3A, B**). A stepwise backward regression was then used to refit the model and the Akaike Information Criterion (AIC) minimum was selected as the final model (**Supplementary 1**). We screened independent predictors of ICI including ECOG PS, combination of TACE, EHM, log (PLR), log (ALT), log (AFP), Child-Pugh score (**Supplementary 2**), and established nomogram to predict the 1-year and 2-years survival probability of patients (**Figure 3C**). In addition, to facilitate the application of this model, we construct an online tool: https://xiaomili.shinyapps.io/DynNomapp/.

**Figure 4:**
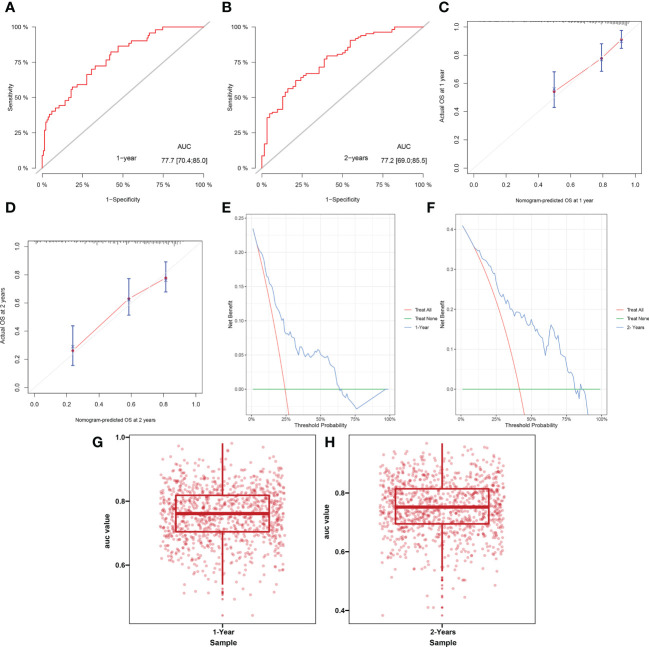
Receiver operating curve **(A, B)**, calibration curve **(C, D)**, decision curve analysis **(E, F)** and cross-validation of AUC **(G, H)** for model predicting survival at 1 year and 2 years.

In all study cohorts, we plotted ROC curve for the model, which had higher AUC value (0.777, 95% CI 0.704 – 0.850) at 1 year and (0.772, 95% CI 0.690 – 0.855) at 2 years, indicating better predictive performance of the nomogram (**Figures 4A, B**). The calibration curve of this model showed good agreement between prediction and observation in survival probability (**Figures 4C, D**). According to the DCA curve, the model predicted a clear net benefit of patients compared to “none” or “full”, and the clinical utility was better (**Figures 4E, F**). In addition, cross-validation showed that the model predicted a median AUC value of 0.7613 (95% CI, 0.7044–0.8185) for 1-year survival and 0.7520 (95% CI, 0.6948–0.8141) for 2-year survival, again demonstrating its discriminant ability (**Figures 4G, H**). Finally, we calculated the C-index for this predictive model, which has a value of 0.7405.

### Risk stratification

**Figure 5:**
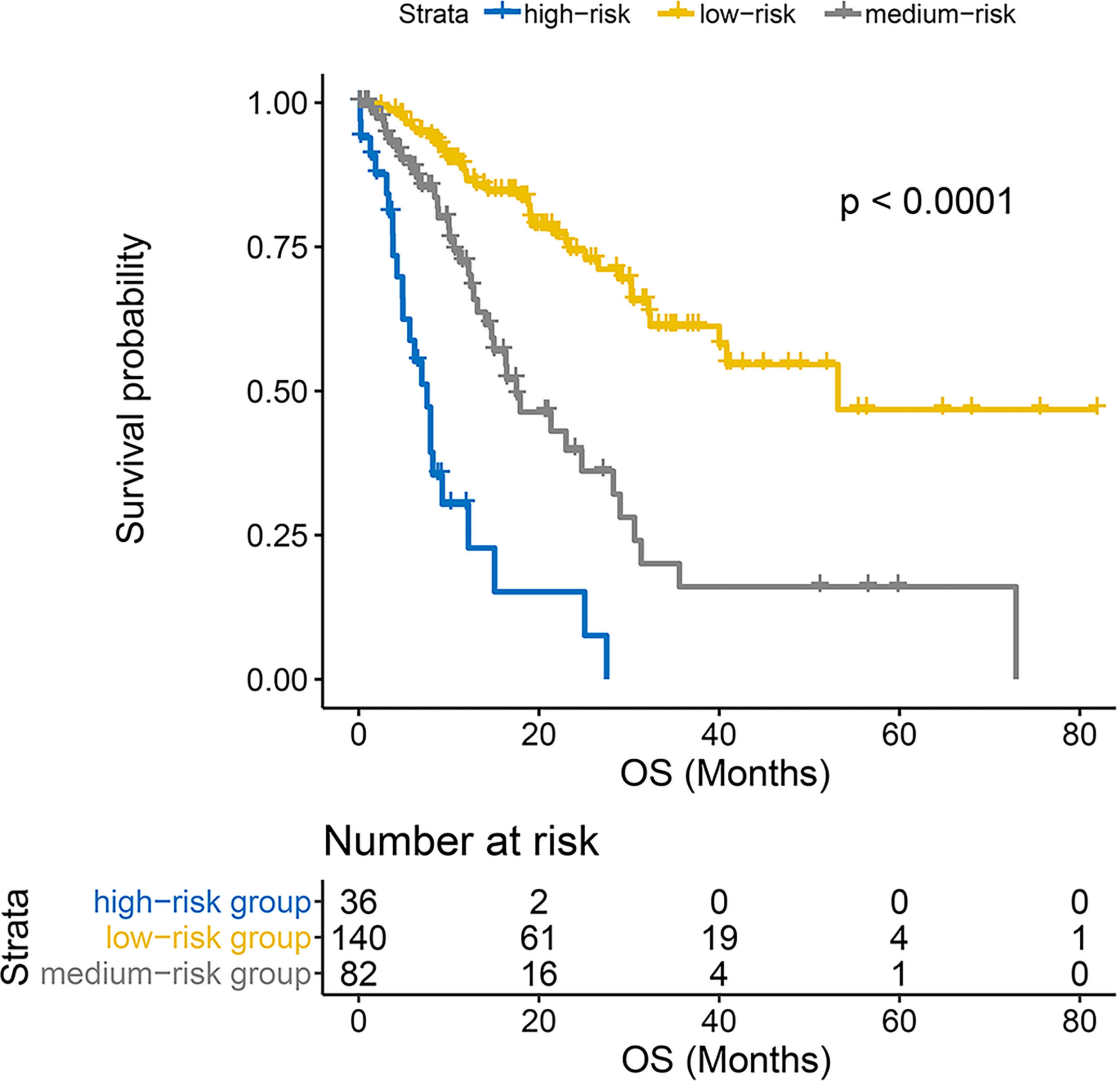
Kaplan-Meier curves for overall survival between the high-, medium- and low-risk groups.

The nomogram integrated seven variables and assigned a score to each predictor, and the total score was calculated according to the actual situation of the patient. And the corresponding survival probability scale was the survival probability of HCC after combination regimen. To visually demonstrate the power of the constructed model, we stratified the total patient score using X-tile software with cutoff values of 182.7 and 240.3. Thus, the study cohort was divided into three risk groups, the high-risk group consisted of 36 patients and 23 deaths, with a mOS of 7.58 (95% CI, 4.9 – 15.1) months; the medium-risk group consisted of 82 patients and 37 deaths, with a mOS of 17.50 (95% CI, 14.1 – 29.0) months; and finally, the low-risk group included 140 patients and 37 deaths, with a mOS of 53.17 (95% CI, 40.0 – not reached (NR)) months. Kaplan-Meier among the three groups was shown in **Figure 5**, with significant differences between groups (P < 0.0001).

## Discussion

The advent of ICIs has greatly improved the prognosis of a variety of solid tumors, including HCC, and has shown synergy in combination with anti-angiogenic agents (16). All patients in our study received a combination of ICIs and anti-angiogenic drugs, and the mOS was as long as 30.2 months, far better than mono-PD-1 inhibitor or mono-TKI in previous studies. Considering the etiological differences between HCC patients worldwide and China, the response to the combination regimen of TKIs plus PD-1 inhibitors will also be different. Therefore, we need to construct a prognostic model of unresectable HCC in Chinese populations, in whom the main etiology was HBV infection, which is different from HCV-, alcohol- or NAFLD- related HCC. However, there is an urgent need in the era of precision medicine to explore reliable predictors and identify subgroups of patients who may benefit from the combination therapy. In this study, we included seven variables and constructed a prognostic model in advanced HCC, with good discrimination, calibration, and applicability. The patient population was divided into three subgroups, with mOS of 53.17, 17.50, and 7.58 months in the low-, medium-, and high- risk groups, respectively, and internal validation again demonstrated its accuracy.

Because there are differences in treatment response to the above combination regimen, the identification and integration of molecular markers is currently a problem to be addressed. Nivolumab tends to improve survival and response in HCC with PD-L1 expression ≥ 1%, but heterogeneity of PD-L1 expression and diversity of detection methods limit its utility (19, 34). High TMB status is a predictive biomarker for second-line treatment with pembrolizumab in advanced solid tumors, but genomic instability makes detection difficult (20, 35). Also, neither PD-L1 expression or TMB were determined to predict the efficacy of mono-PD-1 inhibitors. In the era of comprehensive treatment, it is important to select HCC that may benefit from ICIs combined with TKIs by identifying biomarkers and translate into clinical applications.

As one of the common forms of prediction models, nomograms are widely used in tumor prognosis assessment (36). The mechanism of advanced HCC is complex. Its survival is not only associated with patient characteristics, but also with tumor features including tumor immune microenvironment and genic alterations following treatment. We used Lasso regression and multivariate Cox regression to screen variables, and the potential predictors included ECOG PS, combination of TACE, EHM, PLR, ALT, AFP, and Child-Pugh score.

BCLC stage is a commonly used stage in clinical practice, and ECOG PS, EHM and Child-Pugh score included in our model are important components (37). ECOG PS is an important independent prognostic factor for anti-PD-1 therapy in advanced HCC, which is consistent with the results presented in our study (38). Patients with extrahepatic metastasis were defined as BCLC stage C and the difference in organ tumor microenvironment made their different response. As the most common site of extrahepatic metastasis, lung metastasis has been shown to respond best to treatment in two studies (39, 40). Unfortunately, we did not record specific metastatic sites and further exploration of specific responses in different organs is warranted. Most clinical trials have included only Child-Pugh class A patients to minimize the impact of liver dysfunction on outcomes, whereas we have also investigated Child-Pugh calss B patients. Unlike BCLC stage, we divided according to Child-Pugh score, adding more detail information, thereby improving the accuracy of the model.

Although BCLC stage is widely used in clinical practice, there is a lack of treatment history and inflammatory markers. Other treatment modalities in addition to systemic therapy also affect the survival of HCC, and a small sample study found that TACE tends to delay HCC progression, although no randomized intervention was performed (38). The sequence and time interval between TACE and ICIs are currently inconclusive. The heterogeneity of the tumor microenvironment makes treatment responses different, systemic inflammatory responses play an important role in immune monitoring and treatment assessment (41). NLR and PLR, as the most commonly used inflammatory markers, have been shown to be independent predictors of HCC (42–44). However, no prognostic effect of NLR was found in our study, whereas PLR was included in the model as a continuous variable. High-affinity lymphocytes have antitumor activity and are associated with cancer immune escape (45). Although advanced HCC is often accompanied by thrombocytopenia, platelets can promote the formation of tumor metastasis (46). Therefore, platelet binding to lymphocytes is predictive of HCC with combination regimen, and tends to predict a poor prognosis. In addition, we report the association of other nutritional inflammatory markers (LMR, PNI, and SII) with prognosis, but no significant correlation was found.

Immunotherapy related hepatotoxicity is one of the immune-related AEs and often manifests as an increase in ALT or AST (47). We have found that elevated ALT levels before ICI also predict shorter survival, therefore, management of liver function is challenging. AFP is one of the markers of HCC, and elevated AFP is associated with progression and survival (48). Ramucirumab is used in HCC previously treated with sorafenib and is currently the only agent to select patient populations based on AFP (10). We found that AFP levels were negatively correlated with HCC prognosis treated with ICIs and TKIs and could be used as potential markers to predict patient survival.

The above indicators provide clinicians with direct information on whether to use combination therapy and maximize drug efficacy. Our model contains not only important components of BCLC stage, but also liver function, tumor and inflammatory markers, as well as treatment history. Multiple rather than single prognostic factors were included in the study, which is more comprehensive and optimizes its predictive performance. In addition, the above indicators are routine clinical examinations, non-invasive and easy to obtain.

Limitations of this study are: first, the ICIs and TKIs received by patients are heterogeneous, and no studies have yet demonstrated differences in efficacy among different PD-1 inhibitors; second, there is an inevitable selection bias in the retrospective design, we did not perform stratified analysis according to genes or proteins, and lack predictive analysis of tumor molecular mechanisms for immune prognostic models; finally, the patient population comes from a single center and lacks external validation in other regions, and etiological differences between regions are also non-negligible factors. Additional potential predictors need to be explored prospectively in large multicenter studies in the future, and appropriate subgroups of patients have been selected.

## Conclusion

We constructed a prognostic model for predicting ICIs combined with anti-angiogenic agents for unresectable HCC. The included indicators are comprehensive and simple, and the model has good performance, realizing individualized prediction of patients. The model may predict the efficacy of the combination regimen for advanced HCCs.

## Data availability statement

The raw data supporting the conclusions of this article will be made available by the authors, without undue reservation.

## Ethics statement

The studies involving human participants were reviewed and approved by the ethics review committee of Beijing Ditan Hospital, Capital Medical University. The patients/participants provided their written informed consent to participate in this study.

## Author contributions

Study conception and design, JC and WL. Conception, data collection, assembly of data, project administration, and manuscript preparation, XL and WS. Data collection, data analysis, and manuscript review, XD. All authors contributed to the article and approved the submitted version.

## Acknowledgements

We thank all participants for their endeavor and contribution to this study.

## Conflict of interest

The authors declare that the research was conducted in the absence of any commercial or financial relationships that could be construed as a potential conflict of interest.

## Publisher’s note

All claims expressed in this article are solely those of the authors and do not necessarily represent those of their affiliated organizations, or those of the publisher, the editors and the reviewers. Any product that may be evaluated in this article, or claim that may be made by its manufacturer, is not guaranteed or endorsed by the publisher.
