# Supplementary material for: Prognostic model of immune checkpoint inhibitors combined with anti-angiogenic agents in unresectable hepatocellular carcinoma
Source: Front Immunol. 2022 Dec 1;13:1060051. doi: 10.3389/fimmu.2022.1060051 (PMC9751696; doi:10.3389/fimmu.2022.1060051)
Supplement: Supplementary file 1 [file DataSheet_1.docx]

**Supplementary 1 Multivariate Cox regression with AIC value**

| Model | AIC |
| --- | --- |
| Initial model | 876.27 |
| -PVTT | 871.77 |
| -ALBI | 869.87 |
| -A/G | 868.38 |
| -Number | 867.74 |

**Supplementary 2 The results of multivariate Cox regression**

| Characteristics | Multivariate analysis | |
| --- | --- | --- |
|  | HR(95%CI) | P |
| ECOG (PS1 vs. PS0) | 2.19 (1.42-3.36) | 0.0004 |
| TACE (Yes vs. No) | 0.43 (0.25-0.73) | 0.0021 |
| EHM (Presence vs. Absence) | 1.86 (1.23-2.81) | 0.0033 |
| Log (PLR) | 1.89 (0.83-4.3) | 0.1274 |
| Log (ALT) | 1.96 (0.98-3.9) | 0.0556 |
| Log (AFP) | 1.19 (1.02-1.4) | 0.0309 |
| Child-Pugh Score | 1.48 (1.21-1.81) | 0.0001 |

AIC, akaike information criterion; PVTT, portal vein tumor thrombosis; ALBI, albumin-bilirubin; A/G, albumin/globulin ratio; OS, overall survival; HR(95%CI), hazard ratio(95% confidence interval); ECOG PS, Eastern Cooperative Oncology Group performance status; TACE, trans-arterial chemoembolization; EHM, extrahepatic metastasis; PLR, platelet-to-lymphocyte ratio; ALT, alanine aminotransferase; AFP, alpha-fetoprotein.
